# The AMPK-related kinase NUAK2 suppresses glutathione peroxidase 4 expression and promotes ferroptotic cell death in breast cancer cells

**DOI:** 10.1038/s41420-022-01044-y

**Published:** 2022-05-06

**Authors:** Tanu Singh, Alexander Beatty, Jeffrey R. Peterson

**Affiliations:** Cancer Signaling & Epigenetics Program, Fox Chase Cancer Center, Philadelphia, PA USA

**Keywords:** Cell death, Kinases, Breast cancer

## Abstract

Ferroptosis is a caspase-independent form of regulated cell death strongly linked to the accumulation of reactive lipid hydroperoxides. Lipid hydroperoxides are neutralized in cells by glutathione peroxidase 4 (GPX4) and inhibitors of GPX4 are potent ferroptosis inducers with therapeutic potential in cancer. Here we report that siRNA-mediated silencing of the AMPK-related kinase NUAK2 suppresses cell death by small-molecule inducers of ferroptosis but not apoptosis. Mechanistically we find that NUAK2 suppresses the expression of GPX4 at the RNA level and enhances ferroptosis triggered by GPX4 inhibitors in a manner independent of its kinase activity. *NUAK2* is amplified along with *MDM4* in a subset of breast cancers, particularly the claudin-low subset, suggesting that this may predict vulnerability to GPX4 inhibitors. These findings identify a novel pathway regulating *GPX4* expression as well as ferroptotic sensitivity with potential as a biomarker of breast cancer patients that might respond to GPX4 inhibition as a therapeutic strategy.

## Introduction

Ferroptosis is a form of iron-dependent cell death associated with the oxidation of membrane phospholipids containing unsaturated double bonds into reactive lipid hydroperoxides [1–4]. The biological contexts in which ferroptosis occurs naturally are not well understood but ferroptosis can be pharmacologically induced by small molecules that limit the biosynthesis of the antioxidant glutathione or that directly inhibit glutathione peroxidase 4 (GPX4), the predominant peroxidase for neutralizing toxic lipid hydroperoxides [5]. GPX4 catalyzes the reduction of hydroperoxides into nonreactive lipid alcohols and GPX4 inhibitors are potent inducers of ferroptosis. We previously reported that a subset of triple-negative breast cancer cell lines is dependent on glutathione and GPX4 to avoid ferroptosis [6, 7]. In addition, drug-resistant “persister” cancer cells are also highly dependent on GPX4 for survival [8, 9] and this has led to substantial interest in targeting GPX4 to induce ferroptosis as a therapeutic approach in cancer, though GPX4 inhibitors suitable for clinical use have not yet been reported.

Numerous pathways that mediate sensitivity or resistance to ferroptosis have been identified including the hippo tumor-suppressor pathway effectors YAP and TAZ [10, 11]. YAP/TAZ are transcription regulators that mediate resistance to ferroptosis associated with increased cell density. Their transcriptional activity is regulated, in part, by their partitioning between nuclear and cytoplasmic compartments. Several pathways downstream of YAP/TAZ modulating ferroptosis have been identified [10–15]. For example, TAZ promotes the expression of NADPH oxidases which promote the synthesis of reactive oxygen species and lipid peroxidation under conditions of low cell density [14]. In mesothelioma cells, YAP promotes ferroptosis by driving transcription of the lipid remodeling gene *ACSL4* and the iron transporter *TFRC* [12] and in other contexts via transcription of the E3 ubiquitin ligase *SKP2* [16]. The relative importance of YAP versus TAZ and individual transcriptional targets for ferroptosis is likely to be complex and cell-type specific.

A second pathway known to regulate ferroptosis is mediated by the AMP-activated protein kinase AMPK. AMPK has been shown to either promote or inhibit ferroptosis in different contexts. For example, AMPK-mediated phosphorylation of BECN1 enhances BECN1 binding to the SLC7A11 subunit of the system Xc^-^ cystine transporter, reducing cystine import, resulting in depletion of the cellular redox buffer glutathione and increased lipid peroxidation [17]. AMPK inhibits ferroptosis in other contexts by altering fatty acid synthesis via its substrates acetyl-CoA carboxylase or SREBP [18–20]. Roles in ferroptosis have not yet been reported for the twelve human genes encoding AMPK-related kinases [21], though the NUAK2 kinase has been implicated in nuclear-cytoplasmic trafficking of YAP/TAZ [22, 23]. NUAK2 regulates YAP/TAZ activity in a feed-forward loop in which NUAK2 promotes nuclear translocation of YAP/TAZ by phosphorylating and inhibiting LATS, and nuclear YAP/TAZ in turn enhances *NUAK2* transcription [22, 23], yet whether NUAK2 contributes to promoting ferroptosis downstream of YAP/TAZ is unknown.

Here we identify NUAK2 as an enhancer of ferroptosis. Mechanistically, NUAK2 suppressed *GPX4* expression at the protein and RNA levels. Unexpectedly, we found that NUAK2-mediated *GPX4* suppression was independent of its kinase activity and was not mediated by YAP/TAZ. *NUAK2* expression correlated with sensitivity to GPX4 inhibitors across a variety of human cancer cell lines. We find that *NUAK2* is amplified in a subset of breast cancers and is most highly expressed in the claudin-low subtype. Our findings identify a novel pathway regulating GPX4 and ferroptotic sensitivity and suggest that *NUAK2*-overexpressing patients might respond to therapies targeting GPX4.

## Results

**Fig. 1:**
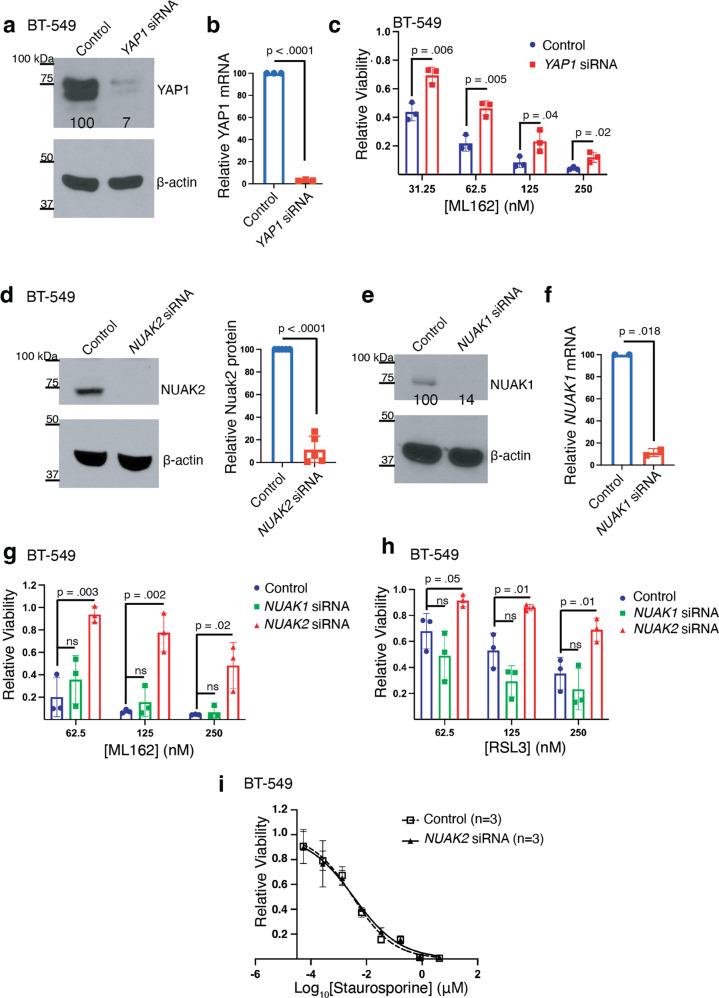
Silencing *NUAK2*, but not *NUAK1*, partially protects TNBC cells from cell death by ferroptosis inducers. **a** Western blot showing YAP1 protein levels in BT-549 cells 72 hours after transfection with a pool of *YAP1*-targeting siRNA or non-targeting siRNA. β-actin is the loading control for this and the subsequent western blots unless otherwise specified. (*n* = 1 independent experiment). Normalized YAP1 protein levels are show beneath the band. **b** Quantitative RT-PCR of *YAP1* mRNA from *YAP1*-siRNA or nontargeting siRNA in BT-549 cells similar to (**a**). *n* = 3 independent experiments and a one sample *t* test was used to test the difference from control *YAP1* mRNA levels (p-value shown above the comparator bar). Error bars, here and in the subsequent panels denote standard deviation centered on the mean. **c** Relative viability of BT-549 cells 72 h after transfection with the pool of *YAP1*-targeting or non-targeting siRNA followed by 48-h treatment with the indicated dose of ML162. *p*-values above comparator bars in this and subsequent panels are from two-sided Student’s *t*-tests unless noted. (*n* = 3 independent experiments). **d** Western blot showing NUAK2 protein level in BT-549 cells 72 h after transfection with a pool of *NUAK2-*targeted siRNA or nontargeting siRNA (*n* = 3 independent experiments). The right panel shows the quantitation of relative NUAK2 protein levels from the 3 experiments and the p-value from a one sample *t* test. **e** Western blot showing NUAK1 protein level in BT-549 cells after *NUAK1* silencing as in (**d**). *n* = 1 **f** Quantitative RT-PCR of *NUAK1* mRNA from *NUAK1*-siRNA or nontargeting siRNA in BT-549 cells similar to (**e**). *n* = 2 independent experiments and a one sample *t* test was used to test the difference from control *NUAK1* mRNA levels. **g** Relative viability of BT-549 cells 72 h after transfection with either *NUAK1*-targeting, *NUAK2*-targeting, or nontargeting siRNA followed by 48-h treatment with indicated dose of (**g**) ML162 or (**h**) RSL3 (*n* = 3 independent experiments). **i** Cell viability dose-response curves in BT-549 cells 72 h after transfection with a pool of *NUAK2*-targeted siRNA or nontargeting siRNA followed by 48 h incubation with the indicated dose of staurosporine, calculated EC_50_ values were 3.2 nM (95% CI, 2–4 nM) for nontargeting siRNA, 3.4 nM (95% CI, 2–5 nM for *NUAK2*-targeted siRNA (*n* = 3 independent experiments).

We examined the pro-ferroptotic role of YAP1 in the TNBC cell line BT-549 by silencing *YAP1* and treating cells with the GPX4 inhibitor ML162. Consistent with YAP1 promoting ferroptosis, an siRNA pool targeting *YAP1*, but not a control siRNA pool, suppressed cell death by the GPX4 inhibitor ML162 (Fig. 1a-c). Similar results (Supplementary Fig. S1a) were obtained on silencing the YAP1 transcriptional co-activator *TEAD4* [24].

We next examined whether NUAK2 and its paralog NUAK1 contribute to ferroptosis in these cells. Silencing of *NUAK2* (Fig. 1d), but not *NUAK1* (Fig. 1e, f), suppressed cell death caused by the structurally distinct GPX4 inhibitors ML162 (Fig. 1g) and RSL3 (Fig. 1h). To determine if NUAK2 affects other forms of cell death such as apoptosis, we next examined BT-549 cells treated with a range of doses of the pan-kinase inhibitor and apoptosis inducer staurosporine [25]. While silencing of *NUAK2* suppressed ferroptosis caused by GPX4 inhibitors, staurosporine exhibited equal potency in control versus *NUAK2*-silenced cells (Fig. 1i; EC_50_ 3.2 nM versus 3.4 nM; Student’s t-test *p* = 0.94).

**Fig. 2:**
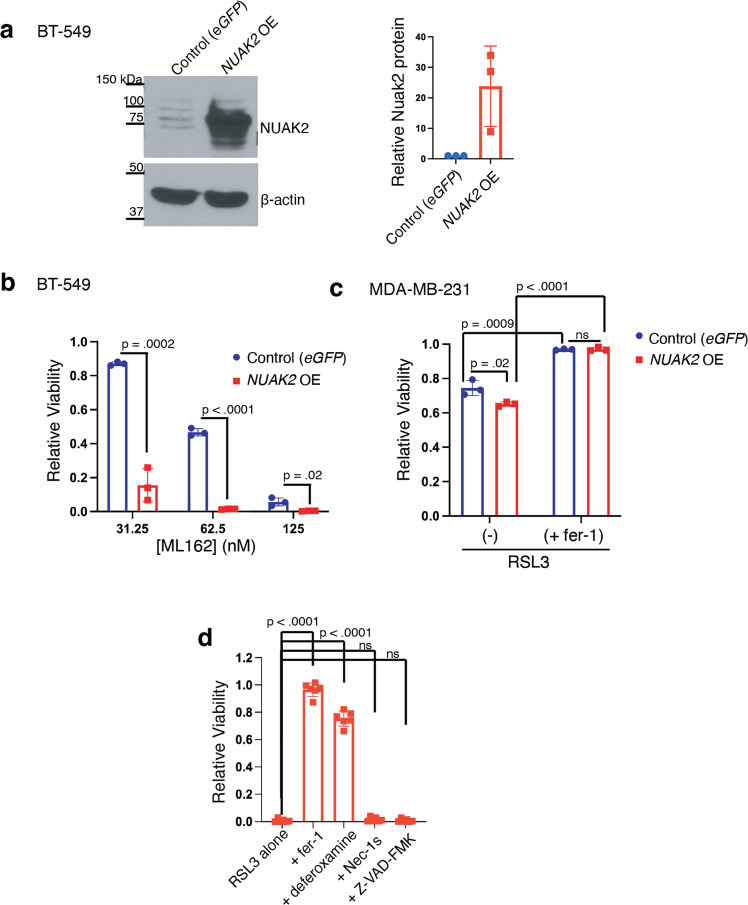
Overexpression of *NUAK2* enhances ferroptosis on GPX4 inhibition. **a** Western blot of BT-549 cells transfected with vectors encoding wild-type NUAK2 or control cell line expressing eGFP (representative of *n* = 3 independent experiments). Right panel shows quantification of relative NUAK2 protein levels from the 3 experiments. Error bars, here and below, denote standard deviation centered on the mean. **b** Relative viability of these cells after 72 h incubation with the indicated dose of ML162 (*n* = 3). *p*-values from two-tailed Student’s *t*-tests are shown here and below. **c** Bar chart showing the relative viability of MDA-MB-231 cells expressing either *eGFP* or *NUAK2* following incubation for 72 h with 62.5 nM RSL3 and co-treatment with either DMSO vehicle (-) or 2 µM ferrostatin-1 (+fer-1) (*n* = 3 independent experiments). **d** Relative viability of NUAK2 overexpressing MDA-MB-231 cells following incubation for 72 h with 125 nM RSL3 and co-treatment with either DMSO vehicle, 2 µM ferrostatin-1, 50 µM deferoxamine, 50 µM Necrostatin-1s or 20 µM Z-VAD-FMK.

To assess whether overexpression of *NUAK2* would have the opposite effect on sensitivity to ferroptosis inducers, we generated a BT-549 cell line stably expressing NUAK2 or eGFP as control (Fig. 2a and Supplementary Fig S2a). *NUAK2*-expressing cell lines exhibited enhanced sensitivity to ML162 (Fig. 2b). Similar potentiation of ML162 toxicity was observed with *NUAK2* expression in another TNBC cell line, MDA-MB-231 (Supplementary Fig. S2b-d), though the effect was less pronounced. This may be because MDA-MB-231 cells exhibit less dependence on glutathione-mediated oxidative defenses than BT-549 cells [6]. NUAK2 also enhanced the toxicity of the GPX4 inhibitor RSL3 (Fig. 2c). The cell death associated with *NUAK2* overexpression in MDA-MB-231 cells was suppressed by ferrostatin-1 (Fig. 2c), consistent with ferroptosis. Furthermore, it was also suppressed by the iron chelator deferoxamine (a ferroptosis inhibitor) but not the RIPK1 inhibitor Necrostatin-1s, nor the caspase inhibitor ZVAD-FMK (Fig. 2d). Together, our findings establish NUAK2 as an enhancer of ferroptosis associated with GPX4 inhibition.

**Fig. 3:**
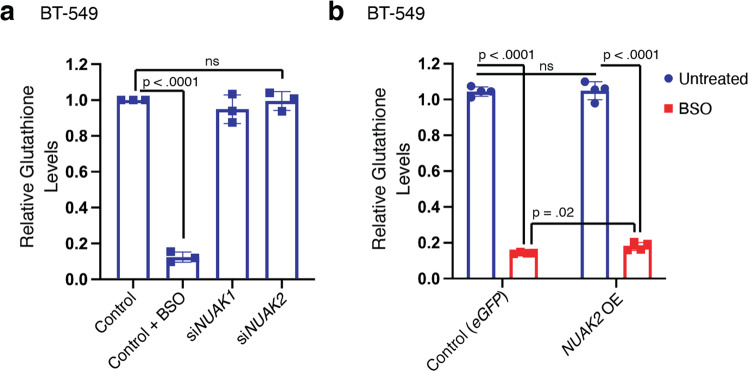
NUAK2 does not enhance ferroptosis by depleting glutathione. **a** Relative glutathione levels (oxidized plus reduced forms) from BT-549 cells 72 h after transfection with either *NUAK1*-targeting, *NUAK2*-targeting, or nontargeting siRNA (control) and 24 h treatment with either vehicle or 20 µM buthionine sulfoximine (BSO) (*n* = 3 independent experiments). **b** Relative glutathione levels from BT-549 cells exogenously expressing NUAK2 or eGFP (controls) 24 h after treatment with either vehicle (untreated) or 20 µM BSO. (*n* = 4 technical replicates). Glutathione levels were normalized to total viable cells (CellTiter-Glo). Error bars denote standard deviation centered on the mean. The numbers above the brackets are *p* values from Student’s *t*-tests (two-sided), ns denotes not significant.

Glutathione is an essential co-factor for GPX4 and depletion of glutathione enhances vulnerability to ferroptosis in numerous contexts. We therefore examined whether NUAK2 affects glutathione levels. As a positive control, buthionine sulfoximine (BSO), an inhibitor of the rate-limiting enzyme of glutathione biosynthesis potently decreased total (reduced plus oxidized) glutathione levels (Fig. 3a). By contrast, silencing of *NUAK1* or *NUAK2* had no effect on glutathione levels compared to non-targeting siRNA controls. Similarly, BT-549 cells overexpressing *NUAK2* retained similar levels of glutathione to cells overexpressing GFP (Fig. 3b; blue bars) and on treatment with BSO, glutathione levels were slightly higher, not lower, in NUAK2-overexpressing cells than in controls. Thus, NUAK2 does not promote ferroptosis by lowering the availability of glutathione.

**Fig. 4:**
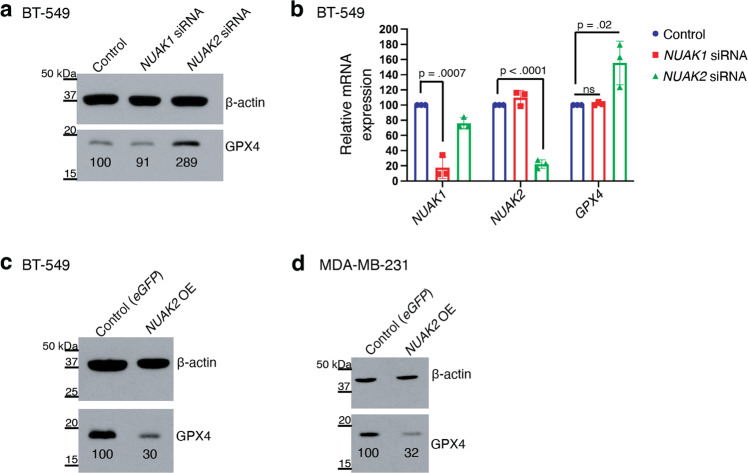
NUAK2 suppresses GPX4 expression at the RNA level. **a** Western blot showing GPX4 protein levels in BT-549 cells 72 h after transfection with a pool of *NUAK1* or *NUAK2*-targeting siRNA or non-targeting siRNA. β-actin is the loading control. (representative of *n* = 3 independent experiments). Relative GPX4 protein levels are show beneath the band. **b** Relative mRNA expression of *NUAK1*, *NUAK2* and *GPX4* by qPCR in BT-549 cells 72 h after transfection with the indicated pool of *NUAK1*, *NUAK2* siRNA or non-targeting siRNA (control) (*n* = 3 independent experiments). Error bars denote standard deviation centered on the mean. The numbers above the brackets are *p* values from Student’s t-tests (two-sided), ns denotes not significant. **c** Western blot showing GPX4 protein levels in (**c**) BT-549 and (**d**) MDA-MB-231 cells, stably over-expressing *NUAK2* compared to the control line expressing *eGFP* (representative of *n* = 2 independent experiments for BT-549 and *n* = 3 for MDA-MB-231). Relative GPX4 protein levels are show beneath the band.

We next tested whether NUAK2 regulates GPX4 levels. BT-549 cells treated with siRNA directed against *NUAK2* had increased GPX4 protein levels compared to non-targeting siRNA as detected by western blotting (Fig. 4a). *NUAK1* siRNA, by contrast, had no effect on GPX4 protein levels. RT-PCR of parallel samples validated that silencing was effective and revealed that *NUAK2* but not *NUAK1* silencing increased *GPX4* mRNA levels, compared to nontargeting siRNA controls (Fig. 4b). Conversely, in *NUAK2*-overexpressing BT-549 cells, GPX4 protein expression was reduced by 60% (Fig. 4c). A similar effect was observed in *NUAK2*-overexpressing MDA-MB-231 cells compared to *eGFP*-expressing controls (Fig. 4d). Thus, *NUAK2* expression negatively regulates GPX4 mRNA and protein levels in these TNBC cell lines.

**Fig. 5:**
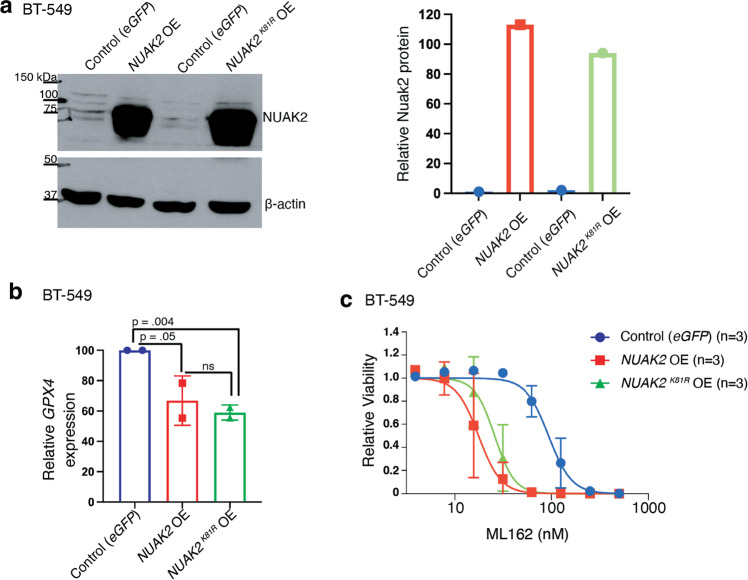
NUAK2 regulation of GPX4 is independent of its kinase activity. **a** Western blot of BT-549 cells transfected with cDNA encoding wild-type *NUAK2* (*NUAK2 OE*) or kinase-dead *NUAK2* (*NUAK2 OE*^*K81R*^) compared to control cells expressing *eGFP*. β-actin is the loading control (representative of *n* = 2 independent experiments). Quantification of normalized NUAK2 protein levels are shown in the right panel. **b** Relative mRNA expression of GPX4 by RT-PCR in BT-549 cells overexpressing either wild-type *NUAK2* or *NUAK2* ^*K81R*^ compared to *eGFP*-expressing controls (*n* = 2 independent experiments). Numbers above the brackets are *p* values from Student’s t-tests (one-sided). **c** Cell viability for these cells treated with the indicated dose of ML162 for 72 h. Calculated EC_50_ values were 94 nM (95% CI, 83−105 nM) for controls (*eGFP*), 18 nM (95% CI, 15–22 nM for *NUAK2 OE* and 26 nM (95% CI, 22-30 nM for *NUAK2*^*K81R*^ *OE* (*n* = 3 independent experiments).

The finding that *NUAK2* overexpression downregulated GPX4 expression indicates that endogenous NUAK2 levels are insufficient for full GPX suppression. This overexpression phenotype, therefore, provided an opportunity to test whether NUAK2’s kinase activity was required for its regulation of GPX4. We mutated lysine 81 (…VAIKSIR…) to arginine to inactivate its catalytic activity [22] and overexpressed it in BT-549 cells at a comparable level to the wildtype *NUAK2*-overexpressing line (Fig. 5a). We then examined *GPX4* expression by RT-PCR in the wild type and mutant *NUAK2* overexpressing lines compared to *eGFP*-expressing controls. *GPX4* mRNA was suppressed to similar extents in both overexpressing lines compared to the *eGFP*-expressing control (Fig. 5b), demonstrating that kinase activity of NUAK2 is dispensable for its suppression of GPX4. Finally, we confirmed that GPX4 suppression by both the wild type (*NUAK2*) and kinase-dead NUAK2 (*NUAK2*^*K81R*^) is associated with enhanced sensitivity to ML162 (Fig. 5c). Kinase dead *NUAK2* expression significantly lowered the EC_50_ of ML162 from 94 nM in control cells to 26 nM in NUAK2 overexpressing cells (Student’s t-test *p* = 0.01), similar to wild type *NUAK2* expressing cells (EC_50_ 18 nM). Thus, NUAK2 plays a role independent of its kinase activity in suppressing GPX4 expression and promoting ferroptosis.

**Fig. 6:**
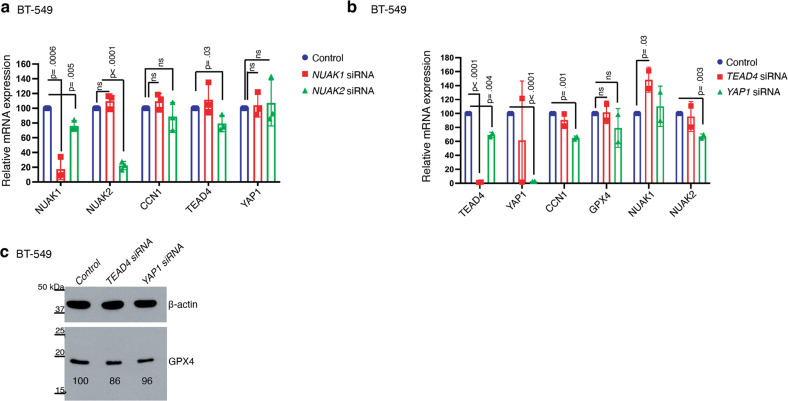
NUAK2 does not affect GPX4 expression via YAP/TAZ. **a** Relative mRNA expression of the indicated genes quantified by qPCR in BT-549 cells 72 h after transfection with the indicated siRNA pool (*n* = 3 independent experiments). Error bars denote standard deviation centered on the mean. *p* values from Student’s t-tests (two-sided) are shown over brackets, ns denotes not significant. **b** qPCR quantitation of the indicated genes in BT-549 cells 72 hours after transfection with the indicated siRNA pool (*n* = 2 independent experiments). Error bars denote standard deviation centered on the mean. The numbers above the brackets are *p* values from Student’s t-tests (one-sided). **c** Western blot showing GPX4 protein levels in BT-549 cells 72 h after silencing of *TEAD4* or *YAP1*. β-actin is the loading control, (*n* = 1). Relative GPX4 protein levels are show beneath the band.

Our data demonstrate that YAP1, TEAD4 and NUAK2 each promote ferroptosis in TNBC cells and that NUAK2 suppresses *GPX4* expression. We therefore hypothesized that NUAK2 suppresses *GPX4* transcription via YAP1/TEAD4. To probe this possibility, we examined mRNA levels of *YAP1*, *TEAD4* and their downstream transcriptional target, *CCN1* (also known as *CYR61*) [26]. Silencing of neither *NUAK1* nor *NUAK2* significantly altered the expression of *YAP1* or *CCN1* in BT-549 cells (Fig. 6a), suggesting that NUAK2 does not alter YAP1 levels or transcription of at least the *CCN1* target gene in BT-549 cells. Interestingly, silencing of *NUAK2* decreased TEAD4 mRNA levels, consistent with literature supporting a role for NUAK2 in promoting YAP/TAZ signaling [22, 23]. To examine whether YAP1 and TEAD4 regulate *GPX4* expression in these cells, we silenced *YAP1* and *TEAD4* but did not observe significant changes in *GPX4* mRNA (Fig. 6b) or protein levels (Fig. 6c). We therefore conclude, contrary to our expectations, that NUAK2 suppression of *GPX4* expression is not mediated by YAP1/TEAD4.

**Fig. 7:**
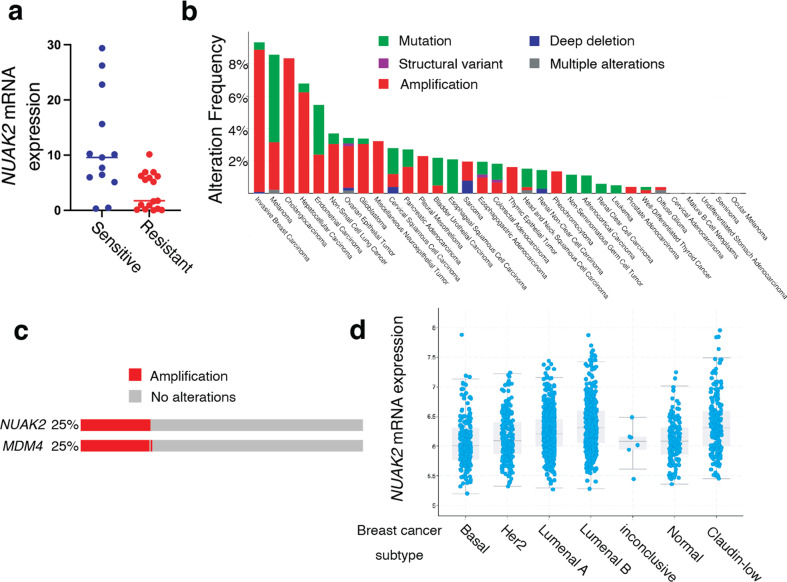
*NUAK2* is frequently amplified in breast cancers, is highly expressed in the claudin-low subtype and is associated with sensitivity to GPX4 inhibition. **a** 100 human cancer cell lines were treated with ML162 or RSL3 at multiple doses for 48 h and cell viability was measured. Cell lines sensitive to GPX4 inhibitors (13 cell lines; blue) were defined based on an area under the dose-response curve (AUC) of >0.15. Resistant cell lines (17 cell lines; red) were defined on the basis of an AUC of 0. *NUAK2* expression was extracted from the Cancer Cell Line Encyclopedia database and is plotted for each GPX4 inhibitor-sensitive or resistant cell line. The *p* value for the between-group comparison (Student’s *t*-test) is *p* < 0.00001. **b** Alterations in the *NUAK2* gene across 10,953 cancer patients from TCGA Pan Cancer Data **c** Oncoprint of 2,173 breast cancer patients from the METABRIC data set [27] showing amplification of *NUAK2* and/or *MDM4*. In (**d**) mRNA expression (microarray) of *NUAK2* in these patients is broken down according to PAM50 breast cancer subtype.

Our results demonstrate an association between *NUAK2* expression and enhanced sensitivity to ferroptosis induced by GPX4 inhibitors in two TNBC cell lines. To test this correlation in additional cell lines, we measured cell viability across a panel of 100 human cancer cell lines treated with up to 1 µM ML162 or RSL3 using the PRISM platform (Broad Institute). We identified GPX4 inhibitor-sensitive and resistant cell lines and compared *NUAK2* expression, from Cancer Cell Line Encyclopedia RNA sequencing data, in these two groups (Fig. 7a). Cell lines most sensitive to GPX4 inhibitors had significantly higher *NUAK2* expression compared to resistant cell lines (*p* < 0.00001, Student’s t-test), supporting an association across cancer types between NUAK2 and GPX4 inhibitor sensitivity.

To assess the clinical relevance of these observations, we examined *NUAK2* alterations across human cancers from The Cancer Genome Atlas (TCGA) data (Fig. 7b). *NUAK2* alterations were most prevalent in breast cancers compared to other cancer types. *NUAK2* alterations were predominantly amplifications (denoted in red in Fig. 7b). Next, we analyzed the METABRIC breast cancer data set of 2,173 patient samples [27] in cBioPortal and found that ~25% had amplification of *NUAK2* (Fig. 7c). *NUAK2* is located in the 1q32 locus along with *MDM4* [28], and *MDM4* was co-amplified with *NUAK2* in almost all *NUAK2*-amplified cases (Fig. 7c). Breast cancer subtype analysis of the METABRIC data revealed that *NUAK2* mRNA expression was highest in the claudin-low subtype (Fig. 7d). MDA-MB-231 and BT-549 cell lines have been classified as claudin low [29]. Together, these observations predict that claudin-low breast cancers will generally exhibit higher *NUAK2* expression and enhanced vulnerability to GPX4 inhibition. This in turn would suggest that claudin-low breast cancers may be susceptible to GPX4 inhibition, should clinical GPX4 inhibitors become available.

## Discussion

Here we identify NUAK2 as a new regulator of ferroptotic sensitivity. Unlike its relative, AMPK, which modulates ferroptosis vulnerability in part by regulating lipid metabolism, we find that NUAK2 reduces mRNA levels of the antiferroptotic enzyme GPX4. Interestingly, *NUAK2* expression was previously reported to be upregulated by a variety of stresses including oxidative stress or an increase in the cellular AMP/ATP ratio [30]. One possibility is that NUAK2 may enforce ferroptotic cell death under conditions of overwhelming cellular stress. Other members of the AMPK-related kinase family have also been implicated oxidative stress [31], perhaps suggesting a general role of this family in cellular responses to oxidative stress.

We found that NUAK2, but not NUAK1, regulated *GPX4* expression and ferroptotic sensitivity; a surprising finding given that they share more than 60% sequence identity [32]. While these related kinases share some substrates, such as MYPT1 and LATS, other substrates are distinct [33], pointing to at least partly distinct functions. Indeed, NUAK1 and NUAK2 exhibit opposing activities in TGFβ signaling [34], supporting nonredundant roles. Interestingly, NUAK2 was found to enhance, while NUAK1 suppressed, TGFβ signaling in this study. TGFβ promotes cell conversion from an epithelial to a mesenchymal state and this transition has been associated with enhanced vulnerability to ferroptosis in cancer cells [9, 35]. Consistent with this, our data suggest a unique role for NUAK2 but not NUAK1 in increasing susceptibility to ferroptosis.

Unexpectedly, we found that NUAK2’s kinase activity is not required for suppressing GPX4 expression nor for enhancing ferroptotic sensitivity. This finding supports a direct link between decreased GPX4 and increasing sensitivity to GPX4 inhibitors. However, the question of exactly how NUAK2 suppresses GPX4 remains open. Since NUAK2 enhancement of YAP nuclear translocation is mediated by NUAK2 phosphorylation and inhibition of LATS1 [22, 23], our finding that NUAK2 kinase activity is not required for suppressing GPX4 (Fig. 5) is consistent with our data that YAP1 does not mediate NUAK2 suppression of GPX4 (Fig. 6). The lack of dependence on its kinase activity suggests a non-canonical signaling function for this kinase that remains to be elucidated. A variety of mechanisms that control *GPX4* transcription in different contexts have been previously reported [36–41] though no clear connections to NUAK2 have been reported.

*NUAK2* expression is upregulated by tumor necrosis factor (TNFα) [42, 43]. TNFα is known as an inducer of apoptotic cell death but our findings suggest that it might also enhance vulnerability to ferroptotic cell death. Indeed, redundancy appears to be common in cell death pathways [44–48], perhaps driven by a strong selective pressure to ensure that unneeded, damaged, or infected cells are eliminated.

A host of metabolic pathways have been previously identified that modulate ferroptotic sensitivity. For example, acyl-CoA synthetase long chain isoforms (ACSLs) either enhance or suppress ferroptosis by modulating the balance of monounsaturated (oxidation resistant) and polyunsaturated (readily oxidized) fatty acids incorporated into cells [7, 49–51]. Likewise, lysophospholipid acyltransferase-mediated incorporation of PUFAs in phospholipids, and lipoxygenases, which catalyze PUFA oxidation, promote ferroptosis [52–56]. Alterations in the expression or activity of anti-oxidant enzymes (e.g. GPX4 or FSP1) or the availability of their small-molecule substrates (glutathione and CoQ10, respectively) similarly modify cell death associated with lipid peroxidation [57–59]. Here we demonstrate a role for a signaling kinase as a novel regulator of this pathway, through its kinase-independent regulation of GPX4 expression. This finding broadens our understanding of the complex mechanisms that modulate the balance between cell viability and oxidative death. Our demonstration that sensitivity to GPX4 inhibitors is correlated with *NUAK2* expression across numerous cancer cell types (Fig. 7a) suggests that *NUAK2* expression may be a potent predictor of vulnerability to GPX4 inhibition.

There in substantial interest in the therapeutic potential of inducing ferroptosis in cancer cells, however clinically tractable reagents to do are still lacking, though this is an area of active research [60–62]. We anticipate that once these have been developed, an important challenge will be identifying cancers most likely to respond to this treatment.

The co-amplification of *NUAK2* and *MDM4* in breast cancer suggests a potential therapeutic application. *MDM4* amplification may be selected for in cancer cells due to its role, shared with its paralog *MDM2*, in inactivating the p53 tumor suppressor. The close proximity of *NUAK2*, which we find is commonly co-amplified with *MDM4* in breast cancer, may fortuitously confer a vulnerability to GPX4 inhibition that could be exploited for therapy.

## Materials and methods

### Cell lines

MDA-MB-231, and BT-549 cell lines were purchased from the American Type Culture Collection (ATCC, Manassas, VA 20110, USA). MDA-MB-231 cell lines were authenticated by short tandem repeat profiling in April 2018. Both cell lines were cultured in RPMI-1640, 10% heat-inactivated fetal bovine serum (FBS), 2 mM supplemental glutamine, and 100 μg/mL penicillin/streptomycin (P/S). Lenti-X 293 T cells (Takara) were cultured in DMEM plus 10% FBS, 2 mM glutamine, and P/S. The FBS content of the medium was increased to 30% during lentiviral production. Cells were cultured in a humidified incubator at 37 °C with 5% CO_2_. Cell lines were periodically tested for *Mycoplasma* contamination using DAPI (4’,6-diamidino-2-phenylindole) to stain DNA.

### Small interfering RNA

For siRNA knockdown experiments, cells were transfected (DharmaFECT 1, Horizon Discovery) with ON_TARGETplus SMART pools (Horizon Discovery) targeting *NUAK1* (Cat# L-004931-01-0005), *NUAK2* (Cat# L-005374-00-0005), *TEAD4* (Cat# L-019570-00-0005), *YAP1* (Cat# L-012200-01-0005) or a nontargeting control pool (Pool #1, D-001810-10-20, Horizon Discovery). The efficiency of mRNA depletion was assessed 72 hours post-transfection using qPCR or western blot (NUAK2 antibody LSBio, Antibody #LS-c331241, lot# 197015, used 1:1000). β-actin antibody (Abcam, ab8227, used 1:5000) was used as a loading control. In experiments to determine suppression of cell toxicity resulting from αESA or ML162 by silencing the expression of individual *NUAK1* and *NUAK2* genes, doses of αESA or ML162 or RSL3 were selected such that there was at least 2–20% remaining viability in cells transfected with non-targeting siRNA following 48 h of treatment.

### Lentivirus-mediated protein expression

Nuak2 or eGFP were expressed using lentivirus-mediated transduction using the pLX304 vector (Addgene plasmid # 25890; http://n2t.net/addgene:25890; RRID:Addgene_25890). *NUAK2* cDNA (GenBank accession BC017306.2; CCSB Human ORFeome Clone Id 7863, Horizon Discovery) was cloned into pLX304 using Gateway cloning (LR reaction, ThermoFisher Scientific). pLX304-CMV-V5-*NUAK2*, pLX304-CMV-V5-*NUAK2*^K81R^ and pLX304-CMV-*eGFP* V5 were co-transfected along with the lentivirus packaging vector (psPAX2; Addgene Plasmid #12260) and envelope vector (pMD2.G; Addgene Plasmid #12259) into Lenti-X 293 T cells (Takara) using X-tremeGENE HP DNA transfection reagent (Roche). Lentiviruses were collected at 48, 72, and 96 h post-transfection and filtered using a 0.22 µm membrane. Lentiviral media was supplemented with 25 mM HEPES (pH 7.4) and stored at −80 °C until use. Target cells were incubated with virus-containing medium and 2 µg/mL polybrene (Sigma Aldrich) for 24 h and then allowed to recover for 24 hours prior to selection with blasticidin (Invivogen, 8 μg/mL for BT-549 and 20 μg/mL MDA-MB-231).

### Mutagenesis

To make the NUAK2^K81R^ point mutant, site-directed mutagenesis by PCR using PfuUltra II HS DNA polymerase was performed using mutagenic primers [5’- CTGGTGGCCATCAGGTCAATC −3’ (forward), 5’- GGTAGTCCAGTTAGGCCTTCC −3’ (reverse)] and pDONR223-*NUAK2* WT (Addgene #23831) as the template. The reaction mixture was transformed into Stbl3 competent cells (ThermoFisher). Miniprep DNA isolated from spectinomycin-resistant colonies was sequenced to identify clones with the desired mutation. A homologous recombination reaction was then performed using LR Clonase II (Life Technologies #11791100) with the pENTR223-*NUAK2* K81R vector and the blasticidin-resistant destination lentiviral vector pLX304-CMV-gateway-V5. The reaction mixture was transformed into Stbl3 competent cells. Ampicillin-resistant colonies were isolated and the mutation confirmed by DNA sequencing.

### Quantitative PCR (qPCR)

Total RNA was isolated using the RNeasy kit (Qiagen) and tested for quality on a Bioanalyzer (Agilent Technologies). RNA concentrations were determined with a NanoDrop spectrophotometer (ThermoFisher Scientific). RNA was reverse transcribed using Moloney murine leukemia virus reverse transcriptase (Ambion- ThermoFisher Scientific) and a mixture of anchored oligo-dT and random decamers (Integrated DNA Technologies). Two reverse-transcription reactions were performed for each sample using either 100 or 25 ng of input RNA. Aliquots of the cDNA were used to measure the expression levels of the genes with the primers, and Power SYBR Green master mix (Applied Biosystems, ThermoFisher Scientific) on a 7900 HT sequence detection system (Applied Biosystems, ThermoFisher Scientific). Cycling conditions were 95 °C, 15 min, followed by 40 (two-step) cycles (95 °C, 15 s; 60 °C, 60 s). Ct (cycle threshold) values were converted to quantities (in arbitrary units) using a standard curve (four points, four-fold dilutions) established with a calibrator sample. The primers (5’ to 3’) used were as follows: *NUAK1* (GGTGTGTTGCTTTACACTCTTG, TATGAGTCCTCGAGCATCTGA), *NUAK2* (CACCTAAACCCTCTGATGCC, CAGTTGACCCACCAGTGAC), *TEAD4* (GTGGTGGAGAAAGTTGAGACA, ACGCTGTTCATCATGTACTTCT), *YAP1* (Taqman assay from LifeTechnologies, Hs00371735_m1), *CCN1* (also known as *CYR61*) (GTGTACAGCAGCCTGAAAAAG, CCGGTATTTCTTCACACTCAAAC) and *GPX4* (ACGTCAAATTCGATATGTTCAGC, AAGTTCCACTTGATGGCATTTC). *36B4* was used as the normalizer (CCCATTCTATCATCAACGGGTACAA, CAGCAAGTGGGAAGGTGTAATCC).

### Cell viability assays and small-molecule treatments

Cells were seeded in 96-well plates (Corning 3917, 3125-6250 cells per well) and treated with compounds 24 hours after plating. Compounds were purchased from Cayman Chemical except for RSL3, purchased from Selleckchem and staurosporine from LC labs. Cell viability was measured using CellTiter-Glo Luminescent Cell Viability Assay (Promega) according to the manufacturer’s instructions. Luminescence was measured on an EnSpire Alpha (Perkin Elmer) using the integrated software package. Data were normalized to vehicle-treated or sensitizing agent-alone controls and sigmoidal dose-response curves were fit using GraphPad Prism (Version 9).

### Glutathione measurements

Total cellular glutathione was quantified using the GSH/GSSG-Glo kit (Promega) according to the instructions provided by the manufacturer. Drug-treated samples were normalized to parallel cell viability measurements using the CellTiter-Glo assay (Promega).

### Antibodies used for western blotting

Western membranes were blocked with 5% powdered milk in tris-buffered saline. Primary antibodies used were against NUAK2 (LSBio, Antibody #LS-c331241, lot# 197015) (1:1000), GPX4 (Abcam, #ab125066, lot# GR3369674-5) (1:1000), β-actin (Abcam, #ab8227, lot# CR3385771-1) (1:5000) or V5 epitope tag (Cell Signaling, (D3H8Q) Rabbit mAb #13202, used 1:2000). Secondary antibodies used were goat-antimouse horseradish peroxidase (ThermoFisher Scientific, #31430, used 1:3000) and goat-antirabbit horseradish peroxidase (ThermoFisher Scientific, #31460, used 1:3000). ImageJ (NIH) was used for the densitometric quantification. Full length original western blots are provided as supplementary materials.

### Analysis of GPX4 inhibitor toxicity in 100 cancer cell lines

100 bar-coded human cancer cell lines were grown as a pool and treated with 0, 10, 40, 110, 330, or 1000 nM ML162 or RSL3 for 48 hours and individual cell lines were quantified by bar code sequencing as described in [63]. Activity area was calculated for each cell line as in [7]. GPX4 inhibitor-sensitive cell lines were defined as those with an AUC of >0.15 for both ML162 and RSL3. GPX4 inhibitor-resistant cells had an AUC of 0 for both ML162 and RSL3. cBioportal was used to extract *NUAK2* expression for the selected cell lines according to the Cancer Cell Line Encyclopedia RNAseq data set (Ghandi et al., Nature 2019). Cell line HEC151 was excluded due to a mutation in *NUAK2*.

### Statistical Analysis

Sample sizes were not determined based on pre-specified effect sizes. Student’s *t*-tests (two-tailed) were used for statistical comparisons unless otherwise noted and the threshold for significance was *p* < 0.05. Data are reported as mean and standard deviation of the indicated number of independent experiments. No samples were excluded from analysis and individual samples are presented in each figure to allow visual confirmation of normality and variance between groups.

## Supplementary information

Supplemental Figs

Original western blots

## Data Availability

All data generated or analyzed during this study are included in this published article (and its supplementary information files) or are available from the corresponding author on reasonable request.
