# Supplementary material for: The AMPK-related kinase NUAK2 suppresses glutathione peroxidase 4 expression and promotes ferroptotic cell death in breast cancer cells
Source: Cell Death Discov. 2022 May 6;8:253. doi: 10.1038/s41420-022-01044-y (PMC9076840; doi:10.1038/s41420-022-01044-y)

Supplementary Figure S1. Relative viability of BT-549 cells 72 h after transfection with siRNA targeting *TEAD4* or non-targeting siRNA (control) followed by 48 hour treatment with the indicated dose of ML162 ($n = 3$ independent experiments). Error bars show standard deviation centered on the mean, and p-values are from two-sided Student's t-tests.

Supplementary Figure 1

a

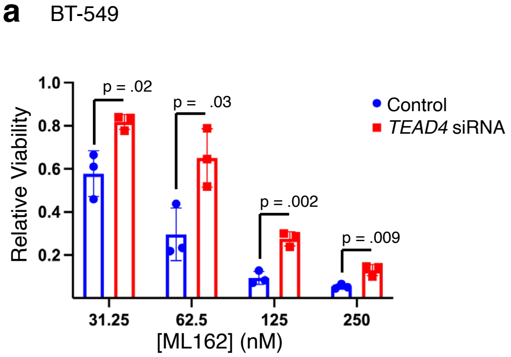

Supplementary Figure S2. (a) Western blot of the V5 epitope tag in BT-549 and (b) MDA-MB-231 cells stably over-expressing the V5-tagged-NUAK2 compared to a control line expressing eGFP (n = 1 independent experiment for both cell lines). (c) Western blot of MDA-MB-231 cells transfected with cDNA encoding wild-type NUAK2 (representative of n = 3 independent experiments). (d) Relative viability of these cells after 72 h incubation with the indicated dose of ML162 (n = 4). Error bars denote standard deviation centered on the mean. p-values from Student's t-tests are shown.

Supplementary Figure 2

a BT-549

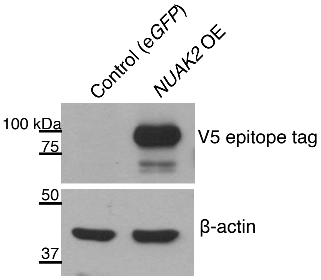

b MDA-MB-231

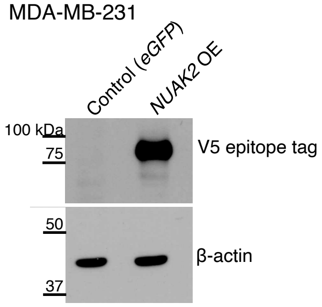

c MDA-MB-231

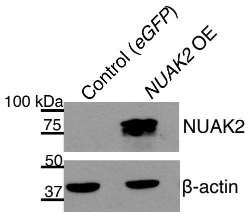

d MDA-MB-231

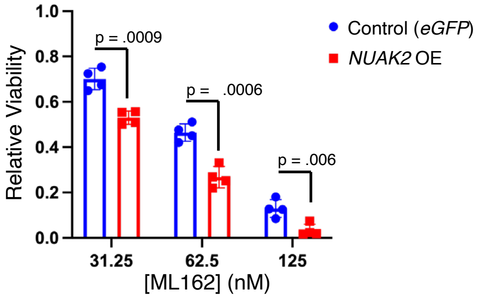

Supplement: Supplementary file 1 — Supplemental Figs [file 41420_2022_1044_MOESM1_ESM.pdf]
