# Supplementary material for: The AMPK-related kinase NUAK2 suppresses glutathione peroxidase 4 expression and promotes ferroptotic cell death in breast cancer cells
Source: Cell Death Discov. 2022 May 6;8:253. doi: 10.1038/s41420-022-01044-y (PMC9076840; doi:10.1038/s41420-022-01044-y)
Supplement: Supplementary file 2 — Original western blots [file 41420_2022_1044_MOESM2_ESM.pdf]

Fig 1a

Western blots

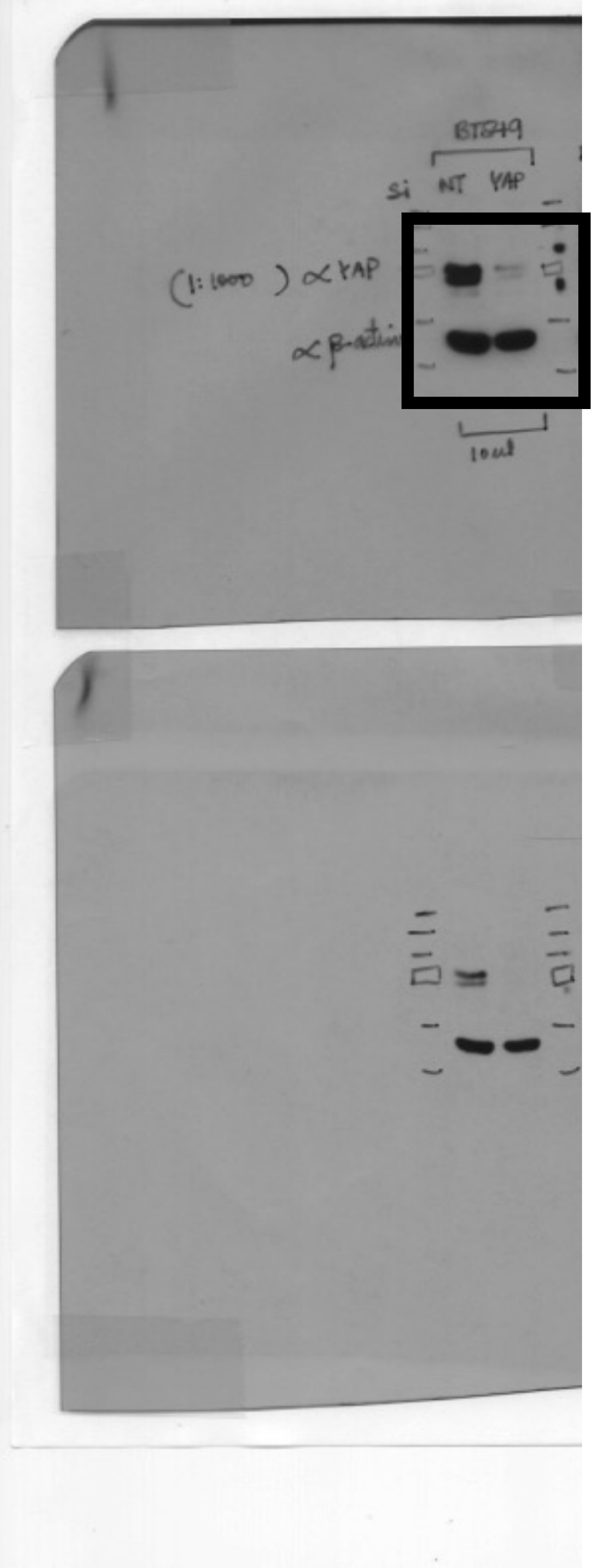

long exposure

anti-YAP1

anti-beta-actin

short exposure

anti-YAP1

anti-beta-actin

Fig 1d
Western blots

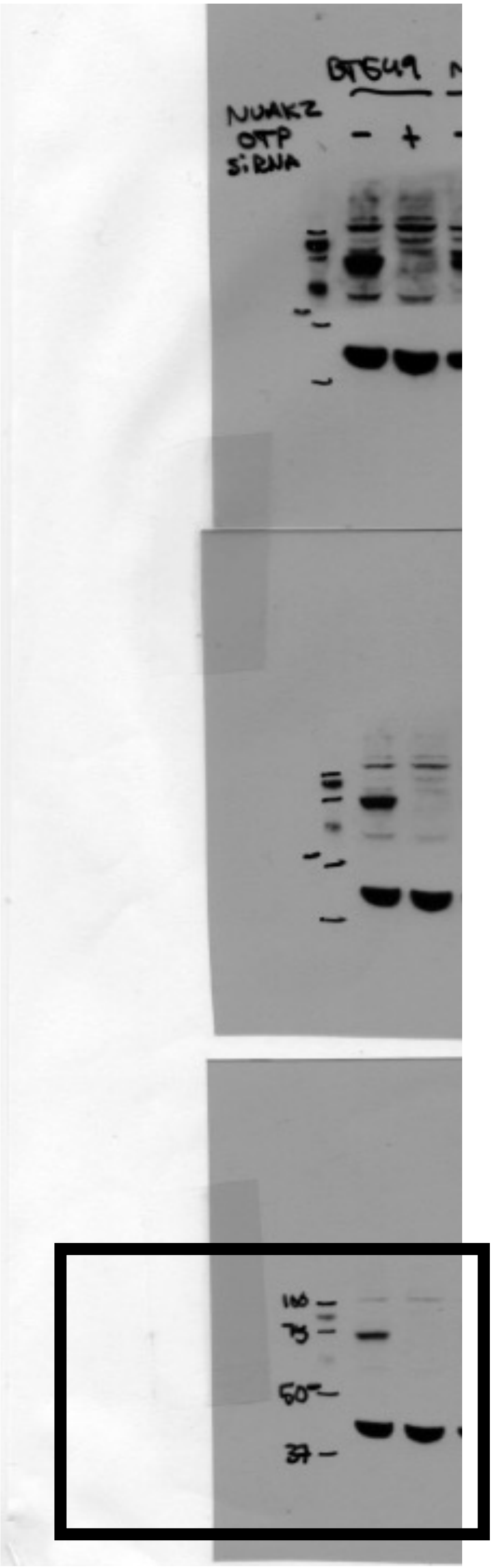

longest exposure

anti-NUAK2

anti-beta-actin

short exposure

anti-NUAK2

anti-beta-actin

shortest exposure
used in the figure

anti-NUAK2

anti-beta-actin

Fig 1e

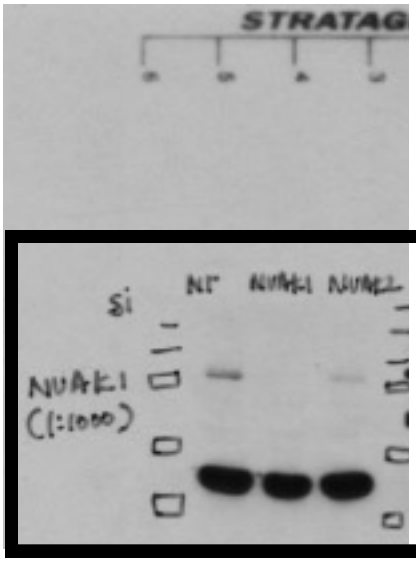

anti-NUAK1 used in the figure

anti-beta-actin

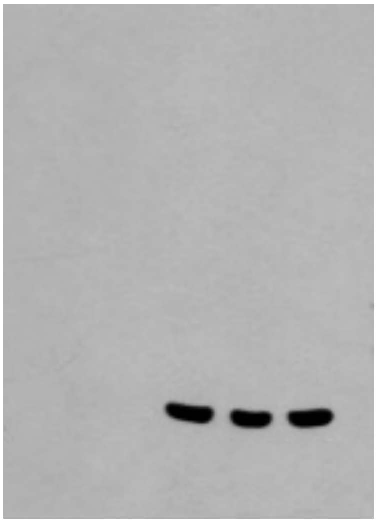

short exposure

anti-beta-actin used in the figure

Fig 2a
Western blots

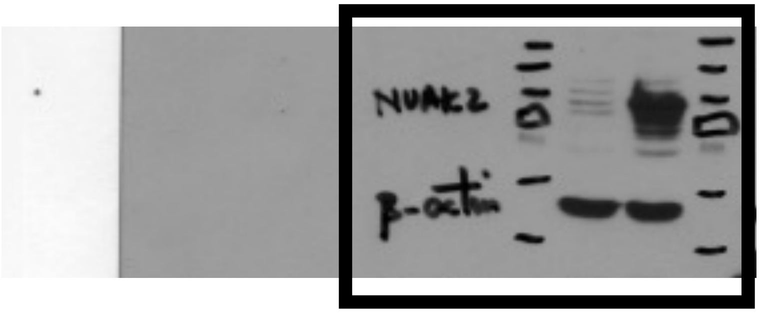

anti-NUAK2

anti-beta-actin

Western blots Fig 4a

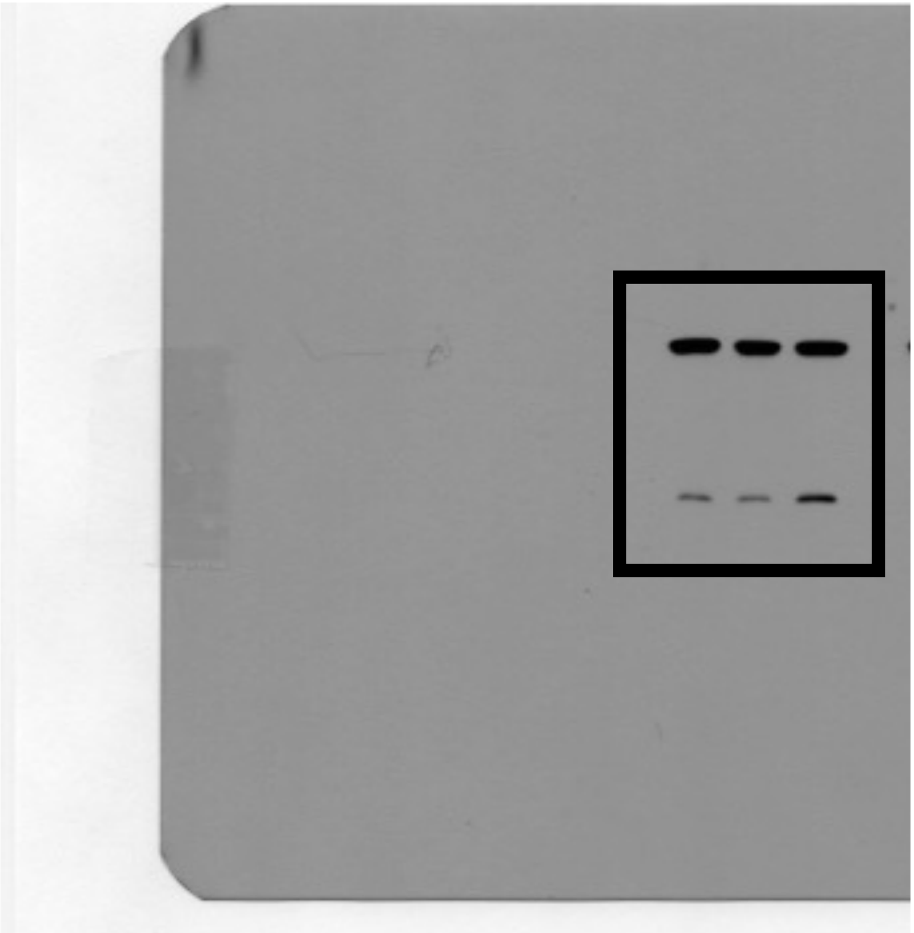

short exposure

anti-beta-actin

anti-GPX4

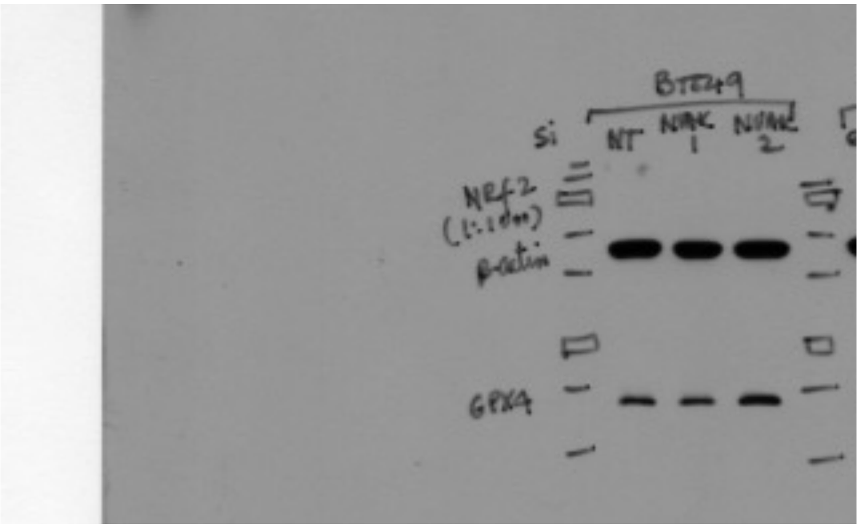

long exposure

anti-beta-actin

anti-GPX4

Western blots

Fig 4c

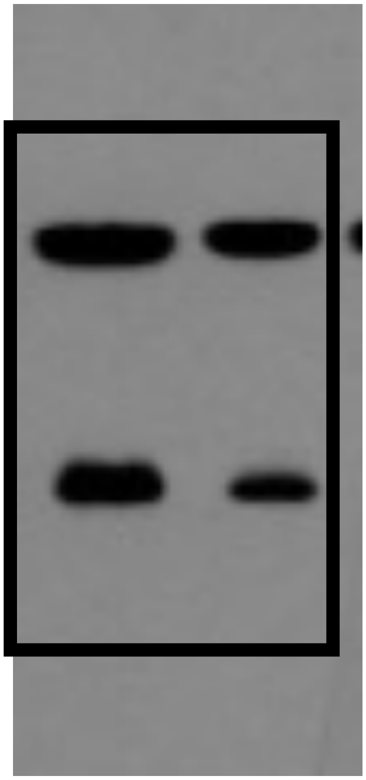

Western blots Fig 4d

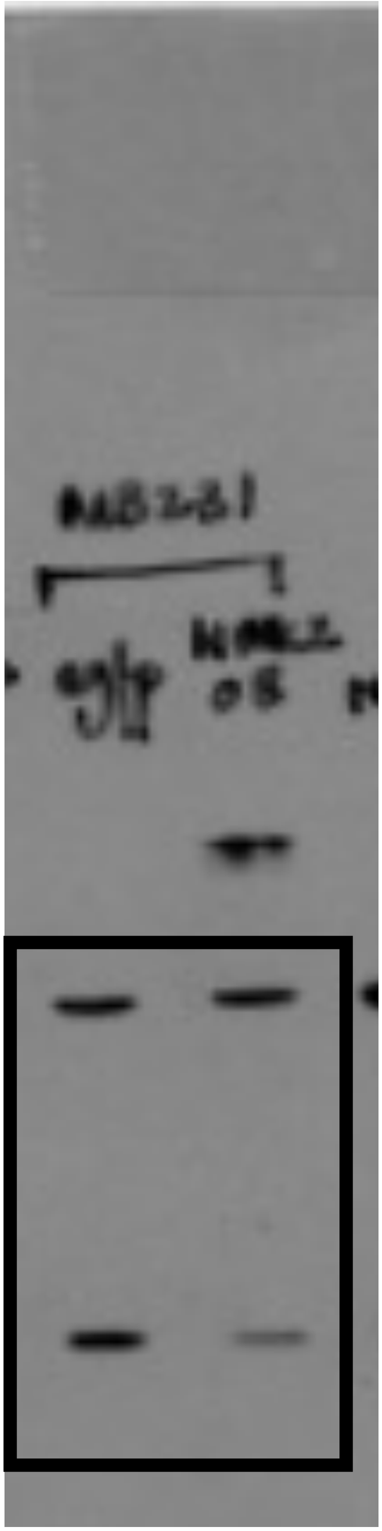

anti-beta-actin

anti-GPX4

Western blots Fig 5a

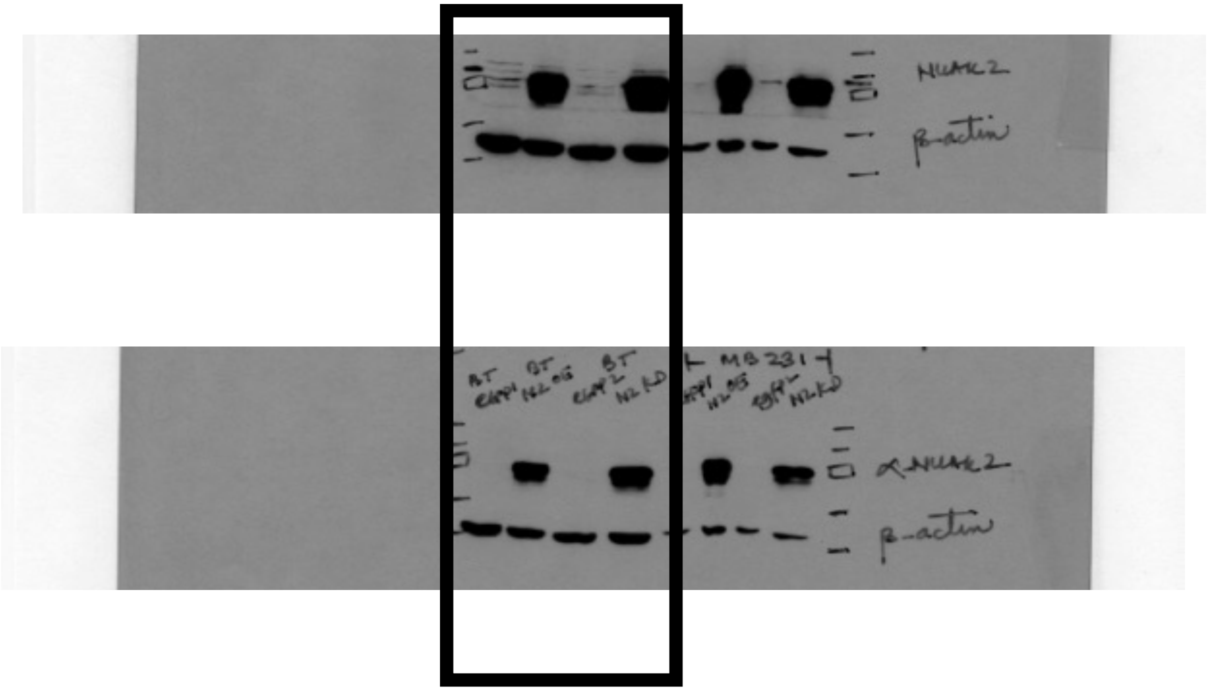

Western blots

Fig 6C

Short exposure

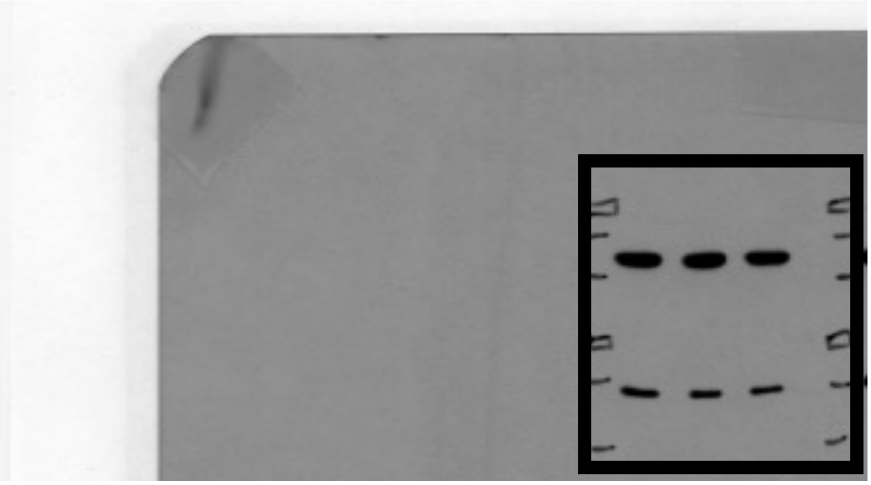

anti-beta-actin

anti-GPX4

Long exposure

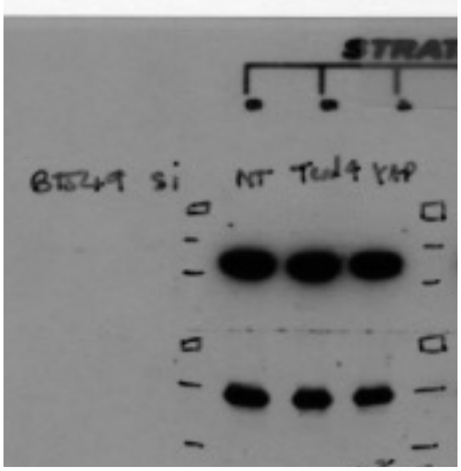

Western blots Fig S2a

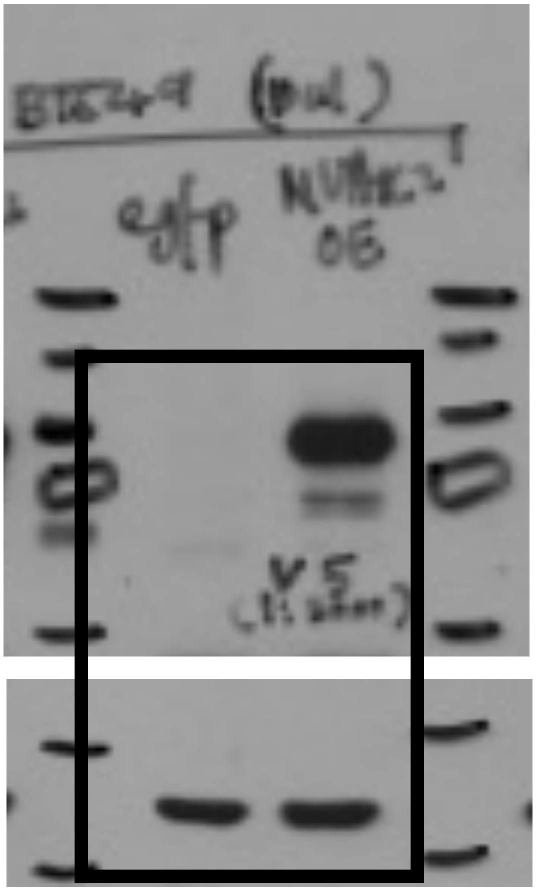

anti-V5

anti-beta-actin

Western blots Fig S2b

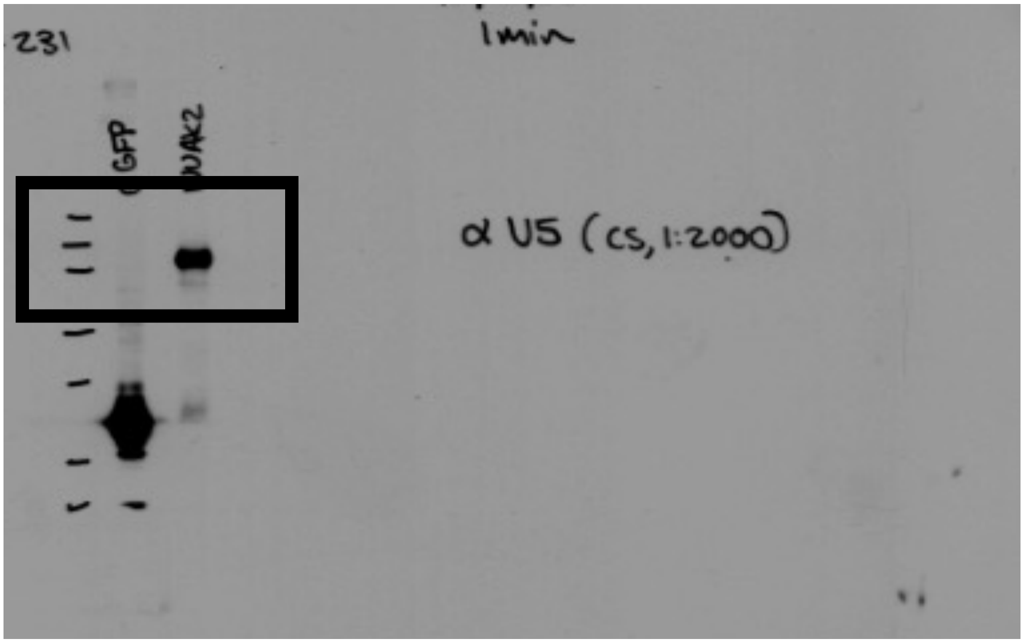

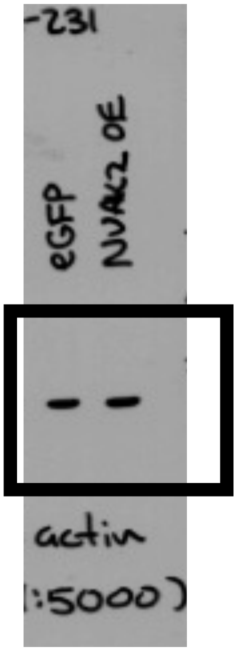

Western blots Fig S2c

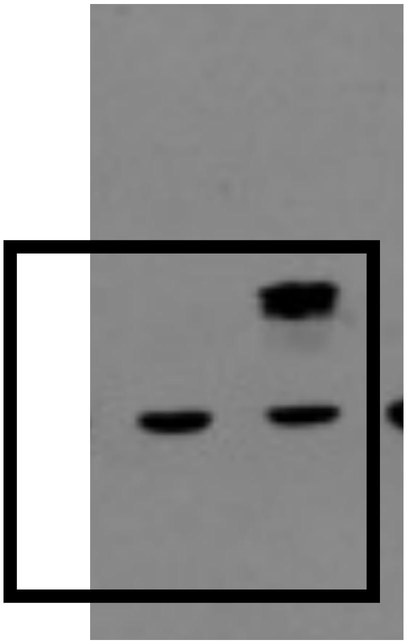

long exposure

anti-NUAK2

anti-beta-actin

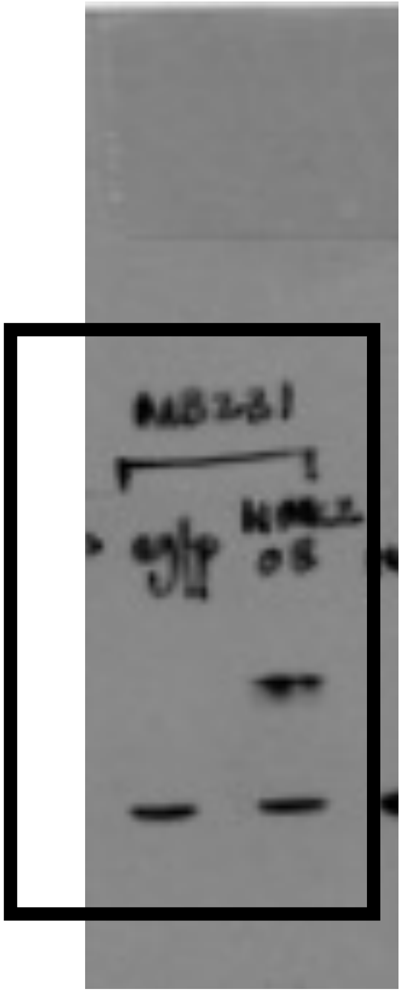

short exposure

anti-NUAK2

anti-beta-actin
